# Are sucking patterns and early spontaneous movements related to later developmental functioning outcomes? A cohort study

**DOI:** 10.1007/s00431-024-05422-9

**Published:** 2024-01-13

**Authors:** Bilge N. Yardımcı-Lokmanoğlu, Numan Demir, Doğan Porsnok, Gülsen Sırtbaş-Işık, Emre Cengiz, Selen Serel-Arslan, Akmer Mutlu

**Affiliations:** 1 Hacettepe University, Faculty of Physical Therapy and Rehabilitation, Developmental and Early Physiotherapy Unit, Ankara, Türkiye; 2 Hacettepe University, Faculty of Physical Therapy and Rehabilitation, Swallowing Disorders Unit, Ankara, Türkiye

**Keywords:** Developmental functioning, Early spontaneous movements, Fidgety movements, General movements, Sucking pattern

## Abstract

Sucking patterns and early spontaneous movements have an important role in the determination of later developmental problems, but the relationship of the two together with long-term outcomes has not been investigated. The objectives of this study were to (i) examine the relationship between sucking patterns using the Neonatal Oral Motor Assessment Scale (NOMAS) and fidgety movements and other movement patterns using detailed General Movements Assessment (GMA), and (ii) investigate the relationship between these early assessment methods and developmental functioning outcomes at later ages. We analyzed the NOMAS from 34 weeks’ postmenstrual age up to 10 weeks post-term and GMA between 9 and 20 weeks post-term age, and the Bayley Scales of Infant and Toddler Development-Third Edition (Bayley-III) was applied for the developmental functioning outcomes to 62 infants (61%, 62/102) between 12 and 42 months of age. Among 102 infants at-risk, 70 (69%) showed a normal sucking pattern, and 85 (83%) had fidgety movements. The median Motor Optimality Score-Revised (MOS-R), as determined by GMA, of all infants was 24. The NOMAS was related to the MOS-R and its subcategories (*p* < 0.05) in all infants at-risk. The NOMAS, MOS-R and its subcategories were also related to cognitive, language, and motor development at later ages according to Bayley-III (*p* < 0.05).

*   Conclusion:* This longitudinal study showed that the quality of sucking patterns, fidgety movements, and MOS-R were related to later developmental functioning, indicating that abnormal sucking patterns, aberrant fidgety movements, and lower MOS-R might predict developmental disorders.

**Table:** 

**What is Known:** *• Sucking patterns and early spontaneous movements in which central pattern generators play an important role are related.* *• Sucking patterns and early spontaneous movements might be used separately to predict developmental outcomes.*	
**What is New:** *• Sucking patterns and early spontaneous movements, when used together, were related to later developmental functioning, including cognitive, language, and motor development in at-risk infants.* *• Predictive value of sucking patterns was lower for each developmental functioning outcome than early spontaneous movements.*

**What is Known:**

*• Sucking patterns and early spontaneous movements in which central pattern generators play an important role are related.*

*• Sucking patterns and early spontaneous movements might be used separately to predict developmental outcomes.*

**What is New:**

*• Sucking patterns and early spontaneous movements, when used together, were related to later developmental functioning, including cognitive, language, and motor development in at-risk infants.*

*• Predictive value of sucking patterns was lower for each developmental functioning outcome than early spontaneous movements.*

## Introduction

Normal sucking requires rhythmical activity and coordination between sucking, swallowing, and respiration [1, 2]. Development of sucking and swallowing ability begins in utero in the first trimester [3, 4]. Sucking and swallowing occur in a 1:1 first month after birth and continue to develop with infant age, including the coordination of sucking, swallowing, and breathing [1]. The sequence of sucking, swallowing, and breathing requires a complex sensorimotor process and neural mechanisms and is controlled by central pattern generators (CPGs) in the brain stem [5–7]. Although CPGs are the basis of this neural mechanism, other brain regions, including the basal ganglia, hypothalamus, cerebellum, amygdala, and tegmental area of the midbrain, the suprabulbar cortical swallowing center, are also involved in this complex control mechanism [5–7].

In addition to controlling CPGs over this sequence, CPGs produce variable movements, including general movements (GMs) [8]. GMs first appear in the fetus and last up to 20 weeks of post-term age [9]. GMs occur in age-specific patterns and are called fidgety movements, which we assessed for this study at between 9 and 20 weeks of post-term age [8]. Fidgety movements are most useful in predicting for neurologic disorder and are described as small movements in all directions with variable acceleration in the neck, trunk, and limbs [10, 11]. At 9 to 20 weeks post-term age, along with fidgety movements, General Movements Assessment (GMA) assesses other movement and postural patterns, and the Motor Optimality Score (MOS, or Motor Optimality Score-Revised/MOS-R) is determined [8, 12]. Fidgety movements have a predictive value for cerebral palsy (CP), with 97% sensitivity and 89% specificity [10, 13, 14], while the MOS was associated with cognitive, language, and motor development in infants born extremely preterm/extremely low birthweight [15, 16], and language performance in typically developing children [17]. Furthermore, MOS was also associated with gross motor functional abilities and activity limitations in children diagnosed with CP [18–20].

Similar to GMA results, the Neonatal Oral Motor Assessment Scale (NOMAS) was also found to be associated with later neurodevelopmental outcomes [10, 13, 14, 21–24]. Tsai et al. [21] reported that infants without brain injury, who had persistent disorganized sucking patterns in the neonatal period, had significantly lower scores in the mental and psychomotor developmental area at 12 months of corrected age. In addition, the NOMAS was associated with cognitive, motor skills, intelligence, and language in children born preterm [22, 24].

A previous study, by Nieuwenhuis et al. [25], revealed that sucking patterns were associated with the quality of fidgety movements and the MOS results in preterm infants, so we know that infants who had better sucking patterns had a higher quality of fidgety movements and MOS. However, it has not to our knowledge been investigated using later developmental functioning.

This study aimed to examine (i) the relationship between sucking patterns using the NOMAS and early spontaneous movements at 9 to 20 weeks post-term age, scored by the detailed GMA, (ii) the relationship between the NOMAS and GMA results and later developmental functioning outcomes using the Bayley Scales of Infant and Toddler Development, Third Edition (Bayley-III).

## Methods

### Participants and study design

**Fig. 1:**
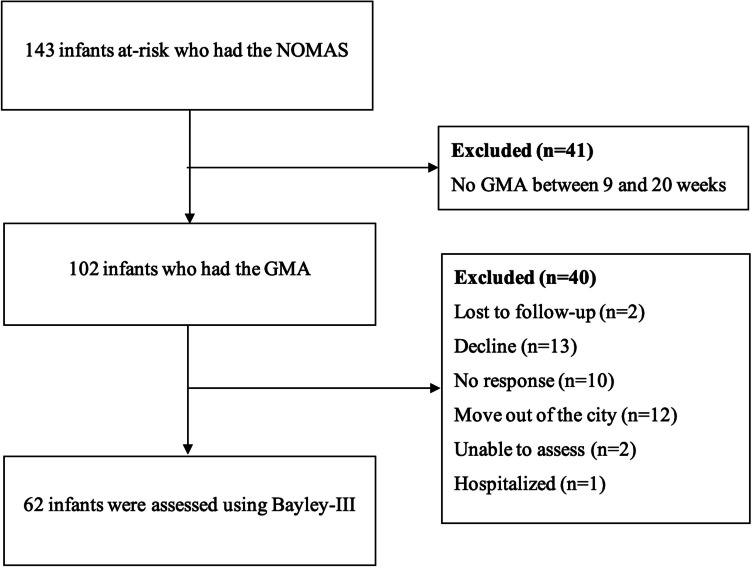
Flowchart of this study

Infants at-risk according to Turkish Neonatal Society guidelines [26], admitted to the Developmental and Early Physiotherapy Unit and the Swallowing Disorders Unit, Faculty of Physical Therapy and Rehabilitation, Hacettepe University, Ankara, Türkiye, participated in this cohort study. These guidelines provide brief recommendations and categorize risk factors [26]. The inclusion criteria were (i) the presence of at least one risk factor in the guidelines, (ii) the NOMAS assessments having been performed up to 10 weeks of corrected age, and (iii) video recordings for GMA having been made between 9 and 20 weeks of corrected age. The infants meeting all these criteria (102 infants) were invited for follow-up assessments between 12 and 42 months of age, and sixty-two infants (61%) were prospectively applied Bayley-III for later developmental functioning outcomes (Fig. 1). Additionally, we created and analyzed the preterm infant group, eliminating other risk factors such as genetic or metabolic disorders. Corrected ages of preterm infants up to two years of age were used. All infants at-risk are followed by several departments of our university until school age to determine whether there is a disorder. We categorized infants as typical development and atypical development based on their diagnosis made by the pediatrician or other specialists.

Written informed consent was obtained from the parents of all infants. This research was approved by the Non-interventional Clinical Research Ethics Board, Hacettepe University (GO 22/303).

## Measurements

### The Neonatal Oral Motor Assessment Scale (NOMAS)

Infants’ sucking patterns were scored in 2-min video recordings using the NOMAS methods by a certified assessor who was blinded to the infants’ clinical history (ND).

The NOMAS consists of 28 items; 14 of which relate to jaw movements, with the remaining 14 related to tongue movements. It is used to diagnose sucking patterns by assessing jaw and tongue movements [27]. The NOMAS categorizes the sucking pattern that underlies poor feeding behavior by assessing the oral-motor components of the tongue and jaw in the neonatal period as normal, disorganized, or dysfunctional [27]. Additionally, da Costa et al. adapted the scoring into five sucking patterns: (i) normal sucking, (ii) disorganized sucking with arrhythmical sucking only, (iii) disorganized sucking with arrhythmical sucking and unable to sustain, (iv) disorganized sucking pattern with arrhythmical sucking, incoordination of suck, swallow, and respiration with or without unable to sustain and (v) dysfunctional sucking pattern [28].

### General Movements Assessment (GMA)

Fidgety movements and other spontaneous movements were assessed with 3-to 5-min video recordings according to Prechtl GMA by two independent assessors who were blinded to the infants’ clinical history (basic and advanced GMA level: AM and BNYL). In cases of disagreement between the scorers (only five recordings, or 4.9%), the video recordings were jointly discussed until consensus on the final score was reached.

The MOS was determined based on the following subscales: (i) temporal organization and quality of fidgety movements, (ii) observed movement patterns other than fidgety movements, (iii) age-adequate movement repertoire, (iv) observed postural patterns, and (v) movement character [8, 20], using the score sheet of the Motor Optimality Score for 3- to 5-Month-Old-Infants–Revised [20]. The fidgety movements subscale is scored 12 (normal), 4 (abnormal), and 1 (absent), while all other subscales are scored 4, 2, or 1, and then the MOS-R results are determined ranging from 5 to 28 points. The maximum MOS-R is determined as 28, indicating the best performance [8].

The interscorer reliability for the MOS was given as the intraclass correlation coefficient, which ranged between 0.80 and 0.94 [29].

### The Bayley Scales of Infant and Toddler Development, Third Edition (Bayley-III)

The Bayley-III scale was applied in children between 12 and 42 months of age by certified assessors (GSI and DP), and composite scores for each domain of Bayley-III were calculated.

The Bayley-III scale is one of the most widely used for assessing the developmental functioning in the early period after birth. It consists of the three domains: Cognitive, Language (Receptive and Expressive), and Motor (Fine and Gross). The raw score of each of the three domains is converted into a scaled score, and then composite scores are calculated for cognitive, language, and motor scales [30].

### Statistical analysis

The SPSS package for Macintosh, version 25.0 (SPSS Inc, Chicago, IL, USA) was performed for statistical analysis. Nonparametric tests were conducted when the variables were not normally distributed. A *p*-value of less than 0.05 was considered statistically significant for all tests. A Bonferroni correction was calculated to adjust for multiple comparisons. The data and assessment results of the groups were compared using the Pearson chi-squared test for categorical variables (e.g., MOS-R subcategories) and the Mann–Whitney U test for continuous variables (e.g., MOS-R). For the analyses of relationships, we used the Spearman correlation coefficient. The value of the correlation coefficient was classified as 0.90 to 1.00 very high correlation, 0.70 to 0.89 high correlation, 0.50 to 0.69 moderate correlation, 0.30 to 0.49 low correlation, and 0.00 to 0.29 little if any correlation [31]. Univariate linear regression analyses were performed to examine the relationship of potential predictors with Bayley-III results. For univariate and multiple linear regression analysis, we classified the NOMAS and fidgety movements as dichotomous (normal and suboptimal). Before the multiple linear regression analysis, we checked assumptions for the multiple linear regression, such as not having multicollinearity problems, no significant outliers, approximately normally distributed in the residuals (errors), and homoscedasticity, and appropriate dependent and independent variables were chosen. Any variable with a variance inflation factor (VIF) that exceeded 4 was excluded from the multiple linear regression model because of the multicollinearity problem.

## Results

We included 102 infants at-risk for whom data on sucking patterns was available from 34 weeks post-menstrual age up to 10 weeks post-term age, and used available data on spontaneous movements in those between 9 and 20 weeks post-term age. Sixty-two infants (61%) were assessed for developmental functioning between 12 and 42 months of age. Additionally, 77 preterm infants whose all-risk factors such as genetic and metabolic were eliminated and categorized for further analysis. The clinical characteristics and assessments age of all infants and only preterm infants are presented in Table 1.

**Table 1:** Clinical characteristics of all infants

	**All Infants (n = 102)**	**Infants who were applied Bayley-III (n = 62)**	**Preterm Infants (77)**	**Preterm Infants who were applied Bayley-III (n = 51)**
**Female/Male, n (%)/n (%)**	54 (53) / 48 (47)	35 (56.5) / 27 (43.5)	46 (60) / 31 (40)	32 (62.7) /19 (37.3)
**Birth Weight, grams, mean (SD)**	2125.51 (805.0)	1876.4 (797.9)	1913.79 (738.2)	1704.26 (684.9)
**Gestational Age, weeks, mean (SD)**	33.67 (3.6)	32.55 (3.7)	32.4 (3.1)	31.67 (3.3)
**Recording Age for NOMAS, (PMA) weeks, mean (SD)**	42.52 (4.3)	42.61 (4.5)	42.05 (4.4)	42.43 (4.7)
**Recording Age for GMA, (PMA) weeks, mean (SD)**	52.96 (2.2)	52.73 (2.1)	53.03 (2.2)	52.76 (2.1)
**Infants’ Risk Factor**				
**Preterm birth, n (%)**	81 (79)	54 (87.1)	77 (100)	51 (100)
**Respiratory distress syndrome (RDS), n (%)**	9 (8.8)	7 (11.3)	9 (12)	7 (13.7)
** Bronchopulmonary dysplasia (BPD), n (%)**	6 (5.9)	5 (8.1)	6 (7.8)	5 (9.8)
** Patent ductus arteriosus (PDA), n (%)**	18 (18)	12 (19.4)	16 (21)	11 (21.6)
** Necrotizing enterocolitis (NEC), n (%)**	2 (2)	0	2 (2.6)	0
** Intrauterine growth retardation (IUGR), n (%)**	24 (24)	14 (22.6)	17 (22)	11 (21.6)
** Small for gestational age (SGA), n (%)**	6 (5.9)	3 (4.8)	3 (3.9)	3 (5.9)
** Hyperbilirubinemia**^**a**^**, n (%)**	11 (11)	4 (6.5)	10 (13)	3 (5.9)
** Periventricular leukomalacia (PVL), ≥ III, n (%)**	1 (1)	1 (1.6)	1 (1.3)	1 (2)
** Intraventricular hemorrhage (IVH), ≥ III, n (%)**	2 (2)	1 (1.6)	2 (2.6)	1 (2)
** Intracranial hemorrhage (ICH), ≥ III, n (%)**	2 (2)	2 (3.2)	2 (2.6)	2 (3.9)
** Hypoxic ischemic encephalopathy (HIE), ≥ II, n (%)**	1 (1)	0	0	0
** Assessment Age for Bayley-III, months, mean (SD) **	-	25.98 (10.2)	-	26.84 (10.6)

*Bayley-III* The Bayley Scales of Infant and Toddler Development, *GMA* general movements assessment, *NOMAS* Neonatal Oral Motor Assessment Scale, *PMA* postmenstrual age, *SD* Standard Deviation

^a^Total serum bilirubin (TSB) value > 12.9 mg/dl

### Typical infants and infants with a diagnosis in later ages

Infants were divided into two groups, typical development and atypical development, according to their diagnoses made by the specialists. Eighty-two infants (80%) had typical development, while some infants (18; 18%) were differently diagnosed at later ages, such as CP (3; 2.9%), global developmental delay (3; 2.9%), Down syndrome (2; 2%), West syndrome (2; 2%), Pierre Robin syndrome (1; 1%), Zellweger syndrome (1; 1%), muscular dystrophy (1; 1%), achondroplasia (1; 1%), epilepsy (1; 1%), language and motor developmental delay (1; 1%), undiagnosed syndrome (1; 1%), and one infant (1%) passed away. In addition to the follow-up assessment, we were not able to learn 2 infants’ (2%) diagnoses.

Eleven infants with atypical development diagnosis were among the 62 infants with Bayley-III results (2 infants with CP, 3 infants with global developmental delay, 1 infant with Down syndrome, 2 infants with West syndrome, 1 infant with muscular dystrophy, 1 infant with achondroplasia and 1 infant with epilepsy). Furthermore, among the preterm infants with Bayley-III, 2 out of 5 infants diagnosed as atypical were CP, and 3 infants had global developmental delay.

### Sucking patterns of infants

Seventy infants (69%) showed a normal sucking pattern in all infants, although 75% preterm infants (58) had a normal sucking pattern. Only 4 (22%) of all atypical infants (18 infants) had a normal sucking pattern, and one of the 6 preterm infants who were diagnosed with CP or developmental delay had a normal sucking pattern. Twenty-eight infants (28%) displayed a disorganized sucking pattern in all infants, and 4 infants (3.9%) had a dysfunctional sucking pattern — these 4 infants were diagnosed atypical at a later age and one of them was preterm (Table 2).

**Table 2:** NOMAS up to 50 week post-menstrual age, and MOS-R and its subcategories at 9–20 post-term weeks of age

		**All Infants (n = 102)**	**Typical Infants (n = 82)**^**d**^	**Atypical Infants (n = 18)**^**d**^	***p***^c^	**Preterm Infants** **(n = 77)**	**Typical Preterm Infants (n = 71)**	**Atypical Preterm Infants (n = 6)**	
**NOMAS**									
**Normal, n (%)**		70 (69)	65 (79)	4 (22)	** < 0.001**^**a**^	58 (75.3)	57 (80)	1 (17)	** < 0.001**^**a**^
**Disorganized: Arrhythmical, n (%)**		20 (20)	14 (17)	5 (28)		12 (15.6)	11 (16)	1 (17)	
**Disorganized: Arrhythmical + unable to sustain, n (%)**		6 (5.9)	3 (3.7)	3 (17)		6 (7.8)	3 (4.2)	3 (50)	
**Disorganized: Arrhythmical + incoordination +/- unable to sustain, n (%)**		2 (2)	0	2 (11)		0	0	0	
**Dysfunctional sucking pattern, n (%)**		4 (3.9)	0	4 (22)		1 (1.3)	0	1 (17)	
**MOS-R and its subcategories**									
**MOS-R**	**Median (range)**	24 (5–28)	25.5 (11–28)	7.5 (5–23)	** < 0.001**^**b**^	24 (5–28)	26 (11–28)	18.5 (5–23)	** < 0.001**^**b**^
	**Optimal (25–28), n (%)**	43 (42)	43 (52)	0	** < 0.001**^**a**^	39 (51)	39 (55)	0	** < 0.001**^**a**^
	**Mildly Reduced (20–24), n (%)**	40 (39)	37 (45)	3 (17)		34 (44)	31 (44)	3 (50)	
	**Moderately Reduced (9–19), n (%)**	8 (7.8)	2 (2.4)	6 (33)		2 (2.6)	1 (1.4)	1 (17)	
	**Severely Reduced (5–8), n (%)**	11 (11)	0	9 (50)		2 (2.6)	0	2 (33)	
**Fidgety Movements**	**Normal, n (%)**	85 (83)	81 (99)	4 (22)	** < 0.001**^**a**^	74 (96)	70 (99)	4 (67)	0.015^a^
	**Abnormal, n (%)**	0	0	0		0	0	0	
	**Absent/Sporadic, n (%)**	17 (17)	1 (1.2)	14 (78)		3 (3.9)	1 (1.4)	2 (33)	
**Observed Movement Patterns**	**N > A, n (%)**	88 (86)	80 (98)	8 (44)	** < 0.001**^**a**^	73 (95)	70 (99)	3 (50)	** < 0.001**^**a**^
	**N = A, n (%)**	5 (4.9)	2 (2.4)	1 (5.6)		2 (2.6)	1 (1.4)	1 (17)	
	**N < A, n (%)**	9 (8.8)	0	9 (50)		2 (2.6)	0	2 (33)	
**Age-adequate Movement Repertoire**	**Age-adequate, n (%)**	53 (52)	51 (62)	2 (11)	** < 0.001**^**a**^	47 (61)	46 (65)	1 (17)	**0.001**^**a**^
	**Reduced, n (%)**	15 (15)	15 (18)	0		13 (17)	13 (18)	0	
	**Absent, n (%)**	34 (33)	16 (20)	16 (89)		17 (22)	12 (17)	5 (83)	
**Observed Postural Patterns**	**N > A, n (%)**	60 (59)	58 (71)	2 (11)	** < 0.001**^**a**^	53 (69)	52 (73)	1 (17)	** < 0.001**^**a**^
	**N = A, n (%)**	19 (8.6)	16 (20)	1 (5.6)		14 (18)	14 (20)	0	
	**N < A, n (%)**	23 (23)	8 (9.8)	15 (83)		10 (13)	5 (7)	5 (83)	
**Movement Character**	**Smooth and Fluent, n (%)**	12 (12)	12 (15)	0	** < 0.001**^**a**^	12 (16)	12 (17)	0	** < 0.001**^**a**^
	**Abnormal, not CS, n (%)**	84 (82)	69 (84)	13 (72)		62 (81)	58 (82)	4 (67)	
	**CS, n (%)**	6 (5.9)	1 (1.2)	5 (28)		3 (3.9)	1 (1.4)	2 (33)	
**Bayley-III**		(n = 62)	(n = 51)	(n = 11)		(n = 51)	(n = 46)	(n = 5)	
**Cognitive Domain, mean ± SD**		95.5 ± 19.0	101.9 ± 12.8	65.9 ± 14.5	** < 0.001**^**b**^	98.6 ± 16.1	102.0 ± 12.3	68.0 ± 16.1	** < 0.001**^**b**^
**Language Domain, mean ± SD**		92.8 ± 18.8	99.2 ± 11.5	62.8 ± 17.0	** < 0.001**^**b**^	95.5 ± 14.9	98.8 ± 11.0	65.0 ± 11.2	** < 0.001**^**b**^
**Motor Domain, mean ± SD**		87.0 ± 18.4	93.9 ± 10.7	55.3 ± 11.9	** < 0.001**^**b**^	89.8 ± 15.5	93.5 ± 10.8	56.2 ± 10.3	** < 0.001**^**b**^

Bold values indicate statistically significant results

*N* > *A* more normal than abnormal patterns, *N* = *A* an equal number of normal and abnormal patterns, *N* < *A* fewer normal than abnormal patterns, *CS* cramped-synchronised movement character, *MOS-R* Motor Optimality Score-revised, *NOMAS* Neonatal Oral Motor Assessment Scale

^a^Pearson Chi-Square test

^b^Mann-Whitney U test

^c^Comparison between Typical and Atypical Infants

^d^Total number of Typical and Atypical Infants were one hundred, because two infants’ outcome were lost

### Early spontaneous movements

The median MOS-R was 24 in all infants and in preterm infant group. Eighty-five infants (83%) had fidgety movements, 17 infants (17%) displayed absent fidgety movements, of which 14 had atypical diagnoses at a later age and two were lost for diagnosis. In preterm infants, 74 (96%) showed fidgety movements and three had no fidgety movements. Although one preterm infant who showed absent fidgety movements was not diagnosed, this infant had a borderline score in Bayley-III and is still followed. The MOS-R and its subcategories’ results are detailed in Table 2.

### Developmental functioning outcomes between 12-and 42-months of age

Sixty-two infants (61%) were assessed by Bayley-III, and 51 of them were preterm. The Bayley-III mean composite scores were 95.48 (SD = 18.98) for the cognitive domain, 92.76 (SD = 18.76) for the language domain, and 87.05 (SD = 18.38) for the motor domain at ages 12 to 42 months in all infants. In preterm infants, the Bayley-III results were 98.63 (SD = 16.13) in the cognitive domain, 95.45 (SD = 14.91) in the language domain, and 89.84 (SD = 15.48) in the motor domain.

Additionally, gestational age was found to be not related to the cognitive domain (Spearman p = 0.06, r = 0.66), the language domain (Spearman p = 0.1, r = 0.46), and the motor domain (Spearman p = 0.1, r = 0.43).

### Relationship between sucking patterns and early spontaneous movements

The quality of sucking patterns was positively related to the MOS-R (p < 0.001, r = 0.42) and all its subcategories in all infants, and the quality of sucking patterns was also related to the MOS-R (p < 0.001, r = 0.40) and all its subcategories in preterm infants (Table 3). While no relationship was found between the quality of sucking patterns and MOS-R and all its subcategories in 82 typical infants (p > 0.05), the quality of sucking patterns was only related to the movement character subcategories of MOS-R (Spearman p = 0.039, r = 0.49) in 18 atypical infants.

**Table 3:** Relationship between early assessment results of sucking patterns and spontaneous movements and developmental functioning outcomes at later age in atypical infants

	**All Infants (n = 102)**												**Preterm Infants (n = 77)**											
	**MOS-R and Its Subcategories**												**MOS-R and Its Subcategories**											
	**MOS-R**		**Fidgety Movements**		**Observed** **Movement Patterns**		**Age-adequate Movement Repertoire**		**ObservedPostural Patterns**		**Movement Character**		**MOS-R**		**Fidgety Movements**		**Observed** **Movement Patterns**		**Age-adequate Movement Repertoire**		**Observed** **Postural Patterns**		**Movement Character**	
	**r**	**p**	**r**	**p**	**r**	**p**	**r**	**p**	**r**	**p**	**r**	**p**	**r**	**p**	**r**	**p**	**r**	**p**	**r**	**p**	**r**	**p**	**r**	**p**
**NOMAS**	**0.42**	** < 0.001**	**0.43**	** < 0.001**	**0.33**	**0.001**	**0.29**	**0.003**	**0.35**	** < 0.001**	**0.34**	**0.001**	**0.40**	** < 0.001**	**0.25**	**0.03**	**0.31**	**0.006**	**0.31**	**0.007**	**0.29**	**0.01**	**0.25**	**0.03**

	**Bayley-III (n = 62)**												**Bayley-III (n = 51)**											
	**Cognitive Domain**				**Language Domain**				**Motor Domain**				**Cognitive Domain**				**Language Domain**				**Motor Domain**			
	**r**		**p**		**r**		**p**		**r**		**p**		**r**		**p**		**r**		**p**		**r**		**p**	
**NOMAS**	**0.36**		**0.004**		**0.42**		**0.001**		**0.48**		** < 0.001**		0.16		0.26		0.25		0.07		**0.36**		**0.01**	
**MOS-R**	**0.53**		** < 0.001**		**0.39**		**0.002**		**0.44**		** < 0.001**		**0.43**		**0.002**		0.26		0.07		**0.30**		**0.04**	
** Fidgety Movements**	**0.55**		** < 0.001**		**0.53**		** < 0.001**		**0.58**		** < 0.001**		**0.33**		**0.02**		**0.38**		**0.006**		**0.38**		**0.006**	
** Observed Movement Patterns**	**0.51**		** < 0.001**		**0.53**		** < 0.001**		**0.50**		** < 0.001**		**0.37**		**0.008**		**0.38**		**0.006**		**0.33**		**0.02**	
** Age-adequate Movement Repertoire**	**0.49**		** < 0.001**		**0.40**		**0.001**		**0.43**		** < 0.001**		**0.42**		**0.002**		**0.32**		**0.02**		**0.31**		**0.03**	
** Observed Postural Patterns**	**0.42**		**0.001**		**0.33**		**0.008**		**0.36**		**0.004**		**0.34**		**0.01**		0.19		0.19		0.27		0.06	
** Movement Character**	**0.48**		** < 0.001**		**0.40**		**0.001**		**0.34**		**0.007**		**0.38**		**0.006**		**0.29**		**0.04**		0.20		0.17	

Bold values indicate statistically significant at the p < 0.05 level

*Bayley-III* The Bayley Scales of Infant and Toddler Development, Third Edition, *MOS-R* Motor Optimality Score-revised, *NOMAS* Neonatal Oral Motor Assessment Scale

### Relationship between the early assessment results of sucking patterns and spontaneous movements and later developmental functioning outcomes

The quality of sucking patterns was positively related to cognitive (p = 0.004, r = 0.36), language (p = 0.001, r = 0.42), and motor (p < 0.001, r = 0.48) developmental functioning in all infants. There was no relationship between sucking patterns and all developmental functioning domains in 51 typical infants (p > 0.05), although there was a relationship between sucking patterns and cognitive (p = 0.039, r = 0.63), language (p = 0.028, r = 0.66), and motor (p = 0.019, r = 0.69) developmental functioning in 11 atypical infants.

There was also a relationship between MOS-R results and cognitive (p < 0.001, r = 0.53), language (p = 0.002, r = 0.39), and motor (p < 0.001, r = 0.44) developmental functioning, and MOS-R subcategories were also related to developmental functioning domains in all infants (62 infants). The relationship results are detailed in Table 3.

In preterm infants (51 infants), the quality of sucking patterns was only related to motor (p = 0.010, r = 0.36) developmental functioning. There was no relationship between sucking patterns and all developmental functioning domains in 46 typical developing preterm infants and 5 atypical developing preterm infants (p > 0.05).

There was also a relationship between MOS-R results and cognitive (p = 0.002, r = 0.43), and motor (p = 0.035, r = 0.30) developmental functioning. Some MOS-R subcategories were also related to developmental functioning domains in preterm infants. The relationship results are detailed in Table 3.

### Univariate and multiple regression analyses

Univariate regression analyses are shown in Table 4. Bayley-III results were predicted by the NOMAS, fidgety movements, MOS-R (p < 0.001).

**Table 4:** Univariate and multiple linear regression of the predictors for the Bayley-III results in all infants

**Bayley-III Domains**	**Variable**	**R**^**2**^	**95% CI for B**	**Beta**	**t**	**p**
**Univariate linear regression (n = 102)**						
**Cognitive Domain**	**NOMAS**	0.18	7.85 to 26.94	0.43	3.65	**0.001**
	**Fidgety movements**	0.43	24.58 to 45.44	0.66	6.71	** < 0.001**
	**MOS-R**	0.50	1.5 to 2.54	0.71	7.81	** < 0.001**
**Language Domain**	**NOMAS**	0.21	9.12 to 27.68	0.46	3.97	** < 0.001**
	**Fidgety movements**	0.45	25.51 to 45.68	0.67	7.06	** < 0.001**
	**MOS-R**	0.52	1.52 to 2.53	0.72	8.02	** < 0.001**
**Motor Domain**	**NOMAS**	0.29	12.8 to 29.99	0.54	4.98	** < 0.001**
	**Fidgety movements**	0.51	27.78 to 46.42	0.72	7.96	** < 0.001**
	**MOS-R**	0.56	1.6 to 2.54	0.75	8.81	** < 0.001**
**Multiple linear regression**^**a**^ **(n = 62)**						
**Dependent variables**	**Independent variables**					
	**Model**^**b**^	**Adjusted R**^**2**^				
**Cognitive Domain**	**NOMAS**	0.49	-6.27 to 11.44	0.06	0.58	0.56
	**MOS-R**		1.31 to 2.54	0.68	6.24	** < 0.001**
**Language Domain**	**NOMAS**	0.51	-4.64 to 12.56	0.1	0.92	0.36
	**MOS-R**		1.28 to 2.47	0.67	6.26	** < 0.001**
**Motor Domain**	**NOMAS**	0.58	-0.14 to 15.48	0.19	1.97	0.05
	**MOS-R**		1.24 to 2.33	0.65	6.55	** < 0.001**

Bold values indicate statistically significant at the p < 0.05 level

*Bayley-III* The Bayley Scales of Infant and Toddler Development, Third Edition, *MOS-R* Motor Optimality Score-revised, *NOMAS* Neonatal Oral Motor Assessment Scale

^a^For multiple linear regression, we included NOMAS and MOS-R as independent variables to predict the cognitive domain, language domain, and motor domain of Bayley-III as dependent variables. Fidgety movements variable with a variance inflation factor (VIF) that exceeded 4 was excluded from the multiple linear regression model because of the multicollinearity problem

^b^Multiple regression equation:

Cognitive domain = 48.67 + 2.58*NOMAS + 1.92*MOS-R

Language domain = 44.68 + 3.96*NOMAS + 1.87*MOS-R

Motor domain = 34.73 + 7.67*NOMAS + 1.78*MOS-R

We used a multiple linear regression model to analyze which predictors (the NOMAS and MOS-R) contributed independently to the Bayley-III domains, and dependent variables for models were cognitive domain, language domain, and motor domain of Bayley-III.

Our models included the NOMAS and MOS-R, which were related to Bayley-III domains, as an independent variable. We classified the NOMAS, as dichotomous (i) “normal” (normal sucking) and (ii) “suboptimal” (disorganized sucking with arrhythmical sucking only, disorganized sucking with arrhythmical sucking and unable to sustain, disorganized sucking pattern with arrhythmical sucking, incoordination with or without unable to sustain, and dysfunctional sucking pattern).

Only the MOS-R remained in the model as a predictor of cognitive domain (adjusted R2 0.49, F(2,59) = 30.298, p < 0.001), language domain (adjusted R2 0.508, F(2,59) = 32.479, p < 0.001), and motor domain (adjusted R2 0.577, F(2,59) = 42.542, p < 0.001).

## Discussion

The NOMAS and GMA are already used to predict neurodevelopmental disorders separately [8–10, 13, 14, 20, 24]. Furthermore, both these assessment methods were shown to be related to later developmental functioning, using Bayley assessment tool [15, 16, 21, 23, 32]. Our study demonstrated that the NOMAS and detailed GMA were related to later developmental functioning outcomes in all infants, in addition to the relationship between each other. Furthermore, the NOMAS and GMA were related to each other in preterm infants, while the NOMAS only related to the motor developmental domain, and some subcategories of detailed GMA were related to different developmental functioning domains.

The rates of disorganized sucking patterns according to the NOMAS were reported as 42.37% in moderately and late infants at 36 to 37 weeks postmenstrual age by Zhang et al. [23] which excluded infants with neurological disorders. Our findings showed a higher incidence of normal sucking patterns in both all infants and preterm infants than a previous study [23]. A first possible explanation for this might be the result of a study by Nieuwenhuis et al. [25] which indicated that the median age at which sucking patterns became normal was 48 weeks postmenstrual age in preterm infants, while we assessed infants ranging from 34 to 50 weeks postmenstrual age. Additionally, da Costa et al. [33] showed that small-for-gestational age preterm infants developed a normal sucking pattern later than appropriate-for-gestational age preterm infants. We included not only preterm infants but also all at-risk infants (even if term infants) in all infants group and the rate of risk factors such as SGA was less in the preterm group, which is the second possible explanation for the higher incidence of normal sucking patterns in the present study.

In 42% of infants, the score was equal to or more than 25 points in the MOS-R in our results, which is considered an optimal range in previous studies [17, 20]. When comparing the findings of previous studies by Peyton et al. [34], and Yuge et al. [35], which reported that aberrant fidgety movements were 24% and 27% respectively in high-risk infants, our percentage of aberrant fidgety movements was low. The low percentage of aberrant fidgety movements in our findings may be a result of different risk factors, as well as various disorders with which some infants were diagnosed. Yuge et al. [35] reported that 14 (38%) infants, of which 72% had aberrant fidgety movements, were diagnosed with different disorders such as CP, severe developmental disorder, or various genetic disorders, while most infants with aberrant fidgety movements were diagnosed with different disorders in our study. On the other hand, the outcomes of our 2 infants with absent fidgety movements were lost. Of the moderate to late preterm infants — the other group in the study of Peyton et al. [34] — 6% had aberrant fidgety movements. This rate was reported as 7.1% in moderate to late preterm infants and 6.7% in all preterm infants [36].

Previously, sucking patterns and early spontaneous movements, including fidgety movements and other MOS subcategories in which CPGs have a crucial role in the control or production, were found to be related to each other in preterm infants [25], which is in line with our study results. During the first months after birth, it has been shown that sucking patterns and early spontaneous movements might separately be early signs of later neurological problems [8, 10, 13, 14, 23, 24]. Firstly, some long-term studies have found a relationship between sucking patterns and neurodevelopmental outcomes, such as Zhang et al. [23] at 6 months in moderately and late preterm infants, and Wolthuis-Stigter et al. [24, 32] at 24 months and at 5 years in preterm infants. Second, Kwong et al. [15, 16] recently found that higher MOS-R were related to better outcomes for development at 2-years of age. Furthermore, Salavati et al. [17, 37, 38] revealed that MOS (or MOS-R) results were related to language development in childhood, cognitive and motor performance at 8 years, and various cognitive domains in young adulthood. In line with the relationships shown previously, we also found a relationship between early signs and cognitive, language, and motor development, besides their relationships with each other. The fact that some of these relationships were absent or had low correlation coefficients in the preterm infant group may be due to the normal development of most preterm infants in this group and better results in sucking patterns and fidgety movements in the early period. However, our findings suggest that the clinical predictive value of early spontaneous movements for developmental disorders in infants was higher than sucking patterns, which might be due to the transition from the fetal circuitries subplate and immature to the progressively developing permanent circuitry cortical plate [39]. This process peaks in the last trimester, and the permanent cortical circuitry of the primary motor, sensory, and visual cortex reaches the final phase around 3 months post-term age [39, 40]. With the permanent cortical circuitry in the cortical plate, GMA had the best prediction value in the fidgety period than previously [8, 39]. In the transient period, the subplate is important for the growth thalamocortical and corticocortical fibers, which it is thought that fidgety movements reorganized by making functional connections between corticospinal fibers and spinal motoneurons [39, 41]. It was also reported that the fidgety movements period, rather than the preterm and writhing periods, had the most predictive power of the later developmental trajectories in young infants [8, 42]. Burger and Louw [43] also revealed that the predictive value of GMs increased from the preterm and/or term period to the fidgety period and reached its the highest value at the fidgety period. Further research is needed to understand GMs in the preterm or writhing periods and the NOMAS in infants on later developmental functioning outcomes. On the other hand, infants’ regulatory skills are better during the feeding when the brainstem nuclei, which control the autonomic nervous system, begin to be regulated by higher brain circuits during the developmental process [44]. For this reason, our results might be affected by the development of the autonomous nervous system, which could be suboptimal in children experiencing pre-perinatal adverse conditions.

This study had a number of possible limitations. The first was that the analyses were based on only single NOMAS and GMA recordings. Second, possible brain injury in infants at-risk including preterm infants might have a risk of broad range of diagnoses, and the assessment age of developmental functioning has a wide range. It is still unclear as to whether the assessment age is related to the Bayley-III results. A few studies included multiple Bayley-III assessments and showed correlations between the longitudinal results of Bayley-III [45, 46]; however, Bayley-III as a predictor of future development is controversial. The final limitation was that we could not apply follow-up Bayley-III assessments on all infants at-risk, and we lost to follow-up 2 infants for developmental outcome.

Among the strengths of our study is the fact that it was longitudinal and the first to describe the relationship between early spontaneous movements and sucking patterns in at-risk infants. Another important strength was the investigation of the predictive value of the NOMAS and GMA for developmental functioning outcomes in infants at-risk together.

In conclusion, the findings of the current study highlight that the NOMAS, MOS-R, and fidgety movements might together be used longitudinally to predict the developmental functioning outcomes of infants at-risk in the earliest period. However, the predictive value of the NOMAS was found to be lower for each developmental functioning outcome than the MOS-R.

## Data Availability

The data that support the findings of this study are available from the corresponding author upon reasonable request. The data are not publicly available due to privacy or ethical restrictions.

## Abbreviations

Bayley-III: Bayley Scales of Infant and Toddler Development-Third Edition
CP: Cerebral palsy
CPGs: Central pattern generators
GMs: General movements
GMA: General movements assessment
MOS: Motor optimality score
MOS-R: Motor optimality score-revised
NOMAS: Neonatal Oral Motor Assessment Scale
